# Effectiveness of a 'Global Postural Reeducation' program for persistent Low Back Pain: a non-randomized controlled trial

**DOI:** 10.1186/1471-2474-11-285

**Published:** 2010-12-16

**Authors:** Francesca Bonetti, Stefania Curti, Stefano Mattioli, Raffaele Mugnai, Carla Vanti, Francesco S Violante, Paolo Pillastrini

**Affiliations:** 1 Section of Occupational Medicine, Department of Internal Medicine, Geriatrics and Nephrology, University of Bologna, Bologna, Italy

## Abstract

**Background:**

The aim of this non-randomized controlled trial was to evaluate the effectiveness of a Global Postural Reeducation (GPR) program as compared to a Stabilization Exercise (SE) program in subjects with persistent low back pain (LBP) at short- and mid-term follow-up (ie. 3 and 6 months).

**Methods:**

According to inclusion and exclusion criteria, 100 patients with a primary complaint of persistent LBP were enrolled in the study: 50 were allocated to the GPR group and 50 to the SE group. Primary outcome measures were Roland and Morris Disability Questionnaire (RMDQ) and Oswestry Disability Index (ODI). Secondary outcome measures were lumbar Visual Analogue Scale (VAS) and Fingertip-to-floor test (FFT). Data were collected at baseline and at 3/6 months by health care professionals unaware of the study. An intention to treat approach was used to analyze participants according to the group to which they were originally assigned.

**Results:**

Of the 100 patients initially included in the study, 78 patients completed the study: 42 in the GPR group and 36 in the SE group. At baseline, the two groups did not differ significantly with respect to gender, age, BMI and outcome measures. Comparing the differences between groups at short- and mid-term follow-up, the GPR group revealed a significant reduction (from baseline) in all outcome measures with respect to the SE group.

The ordered logistic regression model showed an increased likelihood of definitive improvement (reduction from baseline of at least 30% in RMDQ and VAS scores) for the GPR group compared to the SE group (OR 3.9, 95% CI 2.7 to 5.7).

**Conclusions:**

Our findings suggest that a GPR intervention in subjects with persistent LBP induces a greater improvement on pain and disability as compared to a SE program. These results must be confirmed by further studies with higher methodological standards, including randomization, larger sample size, longer follow-up and subgrouping of the LBP subjects.

**Trial registration:**

NCT00789204

## Background

Approximately 70-85% of individuals will experience low back pain (LBP) during their lifetime, and over 80% of them will report recurrent episodes [1]. It is estimated that 80-90% of subjects will recover within 6 weeks, regardless of the type of treatment [1]; however, 5-15% will develop chronic LBP [1]. LBP is defined as pain and discomfort located below the costal margin and above the inferior gluteus folds, with or without referred leg pain [2]. Chronic pain is defined as 'pain that persists beyond the normal time of healing' [3]. Andersson defines it as the persistence of pain for 3 months or longer [4]. Chronic pain seems to be responsible for remarkable direct and indirect costs [5]. As regards the treatment of LBP, exercise therapy appears to be slightly effective for decreasing pain and improving function [6,7]. Exercise therapy encompasses heterogeneous interventions, ranging from aerobic exercise, to muscle strengthening, and flexibility and stretching exercises [8]. To date, dynamic stabilization exercises have been emphasized for improving neuromuscular control, strength, and endurance of specific trunk and pelvic floor muscles that are believed to play an important role in the dynamic stability of the spine [9]. The stabilizing muscles of the spine include all the muscles with intervertebral attachments that are better suited for providing intersegmental stability (multifidus, transversus abdominis, internal oblique), whereas the longer trunk muscles (erector spinae, rectus abdominis) are dedicated to general movement [10]. Some evidence supports the role of stabilization exercises in LBP [11,12]. Moreover, Ferreira et al. obtained slightly better short-term function and perceptions of effects with motor control exercise or spinal manipulative therapy with respect to general exercise [13]. These results are supported by Kumar et al., who concluded that dynamic stabilization exercises are more effective in pain control and functional ability (walking, standing up, and climbing) than electrotherapy (ultrasound and short-wave diathermy) [14].

'Global Postural Reeducation' (GPR) is a physical therapy method developed in France by Philippe-Emmanuel Souchard. This therapeutic approach is based on an integrated idea of the muscular system as formed by muscle chains, which can face shortening resulting from constitutional, behavioral, and psychological factors [15-17]. The aim of GPR is to stretch the shortened muscles using the creep property of viscoelastic tissue and to enhance contraction of the antagonist muscles, thus avoiding postural asymmetry [16]. Although this method is widely employed in countries where Romance languages are spoken, few studies support its theoretical basis and clinical effectiveness. A review of the literature on GPR suggested that this method may be effective for treating some musculoskeletal diseases and disorders such as ankylosing spondylitis, LBP and lumbar disc herniation [18]. More specifically, two randomized controlled trials (RCT) showed that GPR was more effective than analytic stretching and mobilizing exercises in improving clinical and functional measures [19,20]. A treatment that combined oxygen-ozone therapy and GPR appeared to significantly reduce pain and improve the quality of life in patients with lumbar disc herniation [21]. Although some pilot studies were carried out in patients suffering from fibromyalgia [22], patellofemoral pain syndrome [23] and stress urinary incontinence [24], this review pointed out that the available studies do not provide sufficient evidence to draw firm conclusions [18].

More recently, a RCT on female subjects with chronic neck pain showed that conventional static stretching and muscle chain stretching were equally effective in relieving pain and improving both range of motion and quality of life [25]. In another trial, patients with ankylosing spondylitis who underwent GPR treatment appeared to obtain greater benefits on pulmonary function than patients undergoing a conventional exercise program [26].

No previous controlled study investigated the efficacy of GPR in patients with persistent LBP. The aim of this non-randomized controlled trial was to evaluate the effectiveness of a GPR program as compared to Stabilization Exercise (SE) program in subjects with persistent LBP, at short- and mid-term follow-up.

## Methods

The study protocol was registered in the Clinical Trial Registry of the U.S. National Institute of Health (NCT00791596) and was approved by the Independent Ethics Committee in Clinical Research of the University of Bologna.

### Participants

Inclusion criteria were: non-specific LBP in its chronic phase (pain lasting more than 12 weeks), adult age (18 or older). Exclusion criteria were: acute and sub-acute LBP, specific causes of LBP (disc herniation, lumbar stenosis, spinal deformity, fracture, spondylolistesis), central or peripheral neurologic signs, systemic illness (tumour and rheumatologic diseases), psychiatric and mental deficits. Patients who had undergone other physiotherapeutic interventions or surgical operations within 6 months prior to baseline assessment were also excluded.

Five rehabilitation centres were selected for the study. The four smaller centres were assigned to the GPR program and the largest one to the SE program.

From March 2008 to September 2009, all the outpatients with diagnosis of LBP who underwent consultation in one of the five centres were selected by a referent physical therapist, specific for each centre, who was in charge of including or excluding patients. Then, according to inclusion and exclusion criteria, 100 of them were enrolled in the study. Namely, 50 patients from the four smaller rehabilitation centres were assigned to the GPR program, while 50 patients from the largest rehabilitation centre were assigned to the SE program.

All patients gave informed consent to participate in the study, which was conducted according to the provisions of the Helsinki Declaration.

### Interventions

The interventions started immediately after baseline evaluation. Both the GPR and the SE intervention lasted 10 sessions. All sessions were performed in the morning (AM), had a duration of one hour per session, and were conducted with a one-to-one supervision. The frequency was twice weekly for five weeks. Ten physical therapists, with an average experience of 15 years in the GPR approach, were involved in the GPR treatment, whereas 6 physical therapists, with an average experience of 27 years in LBP treatment, carried out the SE program. Each patient of both groups was suggested to repeat the exercises proposed in the last physical therapy session at home every day for 15 minutes.

### Global Postural Reeducation

The GPR involves a series of active gentle movements and postures aimed at realigning joints, stretching shortened muscles and enhancing the contraction of antagonist muscles, thus avoiding postural asymmetry. These therapeutic postures imply an active involvement of the patient. The GPR method includes eight therapeutic postures, lying, sitting or standing, to be held for 15/20 minutes each. Postures can be variously combined during sessions. Postures are chosen on the basis of some parameters, such as amount of pain, load capacity and age of the patient, and muscle chains to be stretched. For the purpose of this study, i.e. to increase the standardization of treatment, the physical therapists proposed only 2 or 3 postures.

In order to make the treatment more uniform and to reduce variability between sessions and physiotherapists, only 2-3 postures among the 8 proposed by the method were used. The postures used are considered the most effective in lengthening the posterior chain, which is usually shortened in patients with LBP.

**Figure 1:**
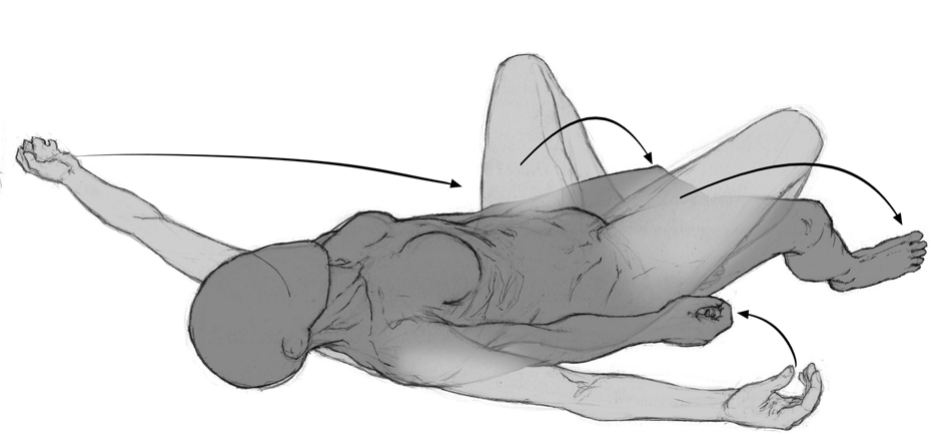
**Positions**. Lying posture with legs extension progression: anterior muscle chain stretching.

**Figure 2:**
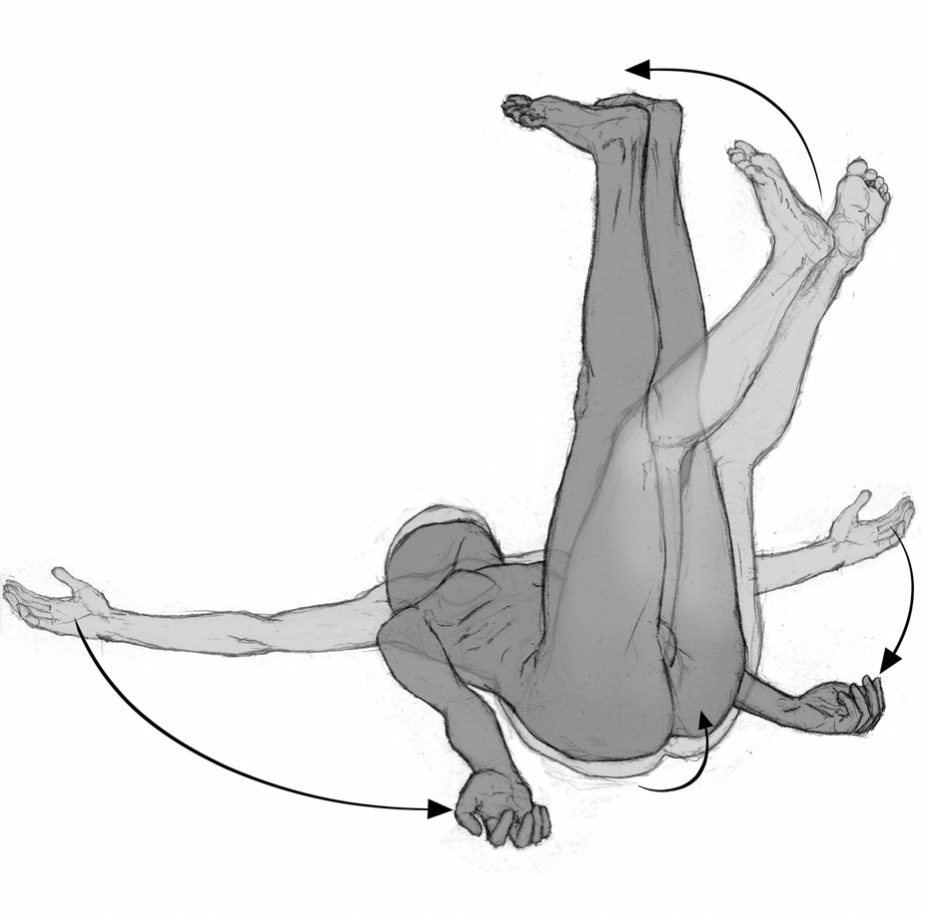
**Positions**. Lying posture with hip joints flexion progression: posterior muscle chain stretching.

**Figure 3:**
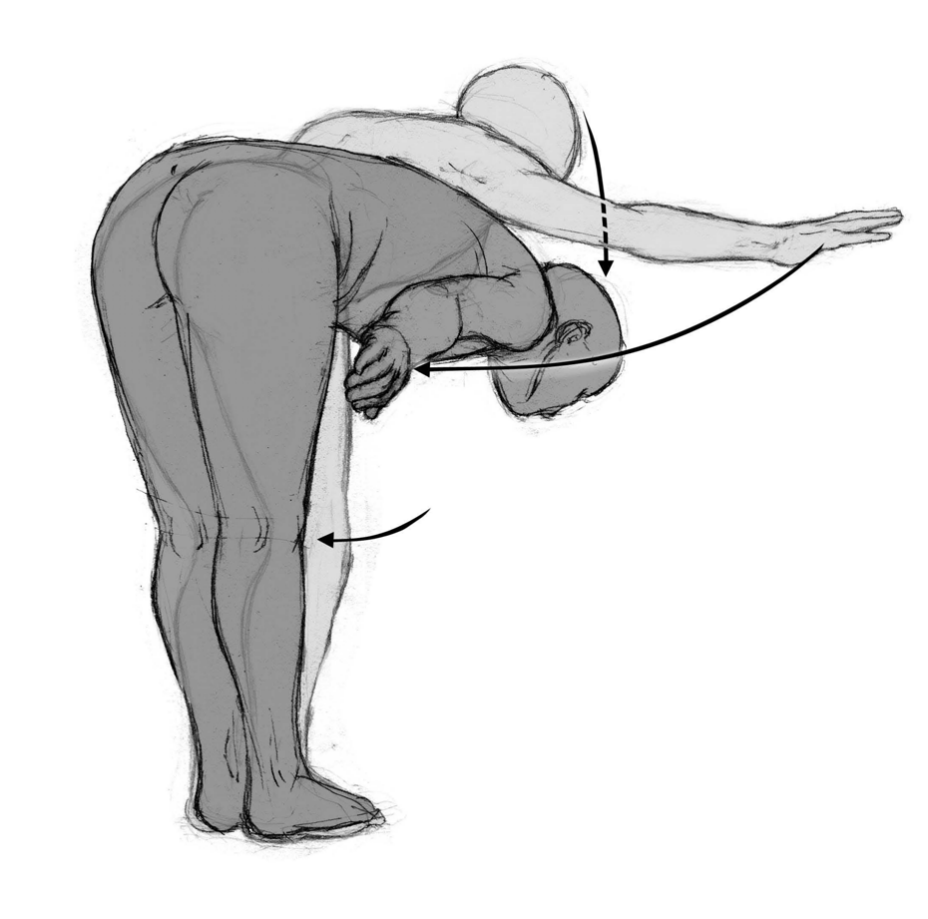
**Positions**. Standing posture with trunk flexion progression: posterior muscle chain loading stretching.

The lying posture with extension of the legs aimed to release the diaphragm muscle and to stretch the anterior muscle chain (diaphragm, pectoralis minor, scalene, sternocleidomastoid, intercostalis, iliopsoas, arm, forearm, and hand flexors) [25]. The patient lied in supine position with the upper limbs abducted about 30° and the forearms supine. Hips were flexed, abducted, and laterally rotated, with foot soles touching each other (Figure 1). Manual traction was applied to the neck in order to align the dorsal and cervical curves of the spinal column, whereas sacral traction was used in order to straighten the lumbar spine. The patient was instructed to spread his hips from the initial position, maintaining the foot soles together in alignment with the body axis. The physical therapist used verbal commands and manual contact to maintain the alignment and make the necessary postural corrections, with the aim of optimizing the stretching and discouraging compensatory movements [27]. The progression was in the direction of extension of the lower limbs and adduction of the upper limbs. The lying posture with flexion of the legs aimed to stretch the posterior chain (upper trapezius, levator scapulae, suboccipital, erector spinae, gluteus maximus, ischiotibial, triceps surae, and foot intrinsic muscles). The initial position was lying with the hip flexed and progression consisted of increasing hip flexion, knee extension, and dorsiflexion of the ankle (Figure 2). The standing posture with flexion of the trunk followed a progression from an upright posture to a bending forward position, while keeping the occiput, the thoracic spine, and the sacrum aligned. This posture was used in order to stretch the posterior chain (Figure 3). Both the lying posture with extension of the legs and the lying posture with flexion of the legs were performed in all patients, whereas the standing posture with flexion of the trunk was performed if allowed by the patient's cooperation, fatigue and pain. In all cases, the total duration of the session was the same. Techniques integrating static and dynamic functions were also employed for about five minutes to give patients the opportunity to experience and use the recovered flexibility in their functional activities (e.g. bending forward, wearing trousers or reaching items at the bottom). Each patient was asked to repeat the exercises at home either in the morning or in the evening according to their capabilities.

### Stabilization Exercise program

The motor control exercise program was based on the treatment approach described in previous publications [12,14]. Exercises were proposed in different combinations and intensities on the basis of the clinical evaluation preliminarily carried out by the physical therapist. Few subjects were not able to perform the exercises proposed in the last phases due both to physical ability and endurance factors.

In the initial phase of the SE program, the physical therapist explained the anatomy of the local stabilizing muscles and how to selectively activate them. The program began with the low-load activation of the local stabilizing muscles isometrically and in minimally loading positions (supine lying, sitting, standing, 4-point kneeling). The patient was instructed to breath normally while activating or holding muscular contraction. Progressively, the holding time and the number of contractions were increased up to 10 contraction repetitions × 10-second duration each. Once the specific pattern of co-activation was achieved in the minimally loading positions and the subjects could comfortably perform 10 contraction repetitions × 10-second duration each, they were asked to perform dynamic functions (activities that required spinal or limb movements) through incorporation of the stabilizing muscles' co-contraction into light functional tasks. Finally, when the patient was able to correctly perform the exercises proposed in the previous step, lumbar stabilization during heavier-load functional tasks (e.g. bridging exercise) and high-speed phasic exercises (e.g. leg cycling) were introduced. Patients were advised to continue with their exercise regimen at home.

### Outcomes

After obtaining a written informed consent from all the participants, basic demographic data (age, gender, BMI), smoking habits, work-related characteristics and educational level as well as duration of symptoms and previous treatment were recorded.

Outcome measurements were collected at baseline and at two follow-up examinations after 3 and 6 months from baseline by health care professionals who were unaware of the study. The 2^nd ^follow-up was performed four months and a half after the end of treatment.

The primary outcome of this study was the perceived level of disability as a result of LBP assessed by the following self-administered evaluation scales: the Roland & Morris Disability Questionnaire (RMDQ) and the Oswestry Disability Index (ODI). The RMDQ is validated in Italy [28], and comprises 24 items in which greater levels of disability are reflected by higher numbers on a 24-point scale [29]. The RMDQ has been shown to yield reliable measurements, which are valid for inferring the level of disability, and to be sensitive to change over time for groups of patients with LBP [30,31]. The ODI, which was used in the Italian version [32], is structured in 10 sections corresponding to different daily activities, each scored on a six-point scale (0-5). Scores of 0-20% indicate minimal disability, 20-40% moderate disability, 40-60% severe disability, 60-80% crippled, 80-100% either bed-bound or exaggerating symptoms [33,34].

Secondary outcome measures included the evaluation of lumbar physical discomfort assessed with a 100 mm Visual Analogue Scale (VAS), whereas mobility of the whole spine and pelvis was evaluated with the Fingertip-to-floor test (FFT). The VAS scores ranged from 0 (no pain) to 100 (the worst possible pain). The VAS has been proved to be reliable and satisfactory in the measurement of pain [35]. As regards the FFT, the subjects stood erect on a 20 cm high platform with shoes removed and feet close together. They were asked to bend forward as far as possible, while maintaining the knees, arms, and fingers fully extended. The vertical distance between the tip of the middle finger and the platform was measured with a supple tape measure and was expressed in centimetres [36]. Decreased distance indicates increased lumbar flexion [37].

### Data analysis

Baseline descriptive statistics were reported for each group regarding personal and work-related characteristics and outcome measures. Continuous data were expressed as means and standard deviations (SD), while categorical data were presented as absolute numbers and percentages (%). In order to compare the baseline characteristics of the GPR and the SE group, the Student's t test was used for continuous variables, whereas categorical variables were assessed using the X^2 ^test or the Fisher's exact test, as appropriate.

Data were analysed in two different ways to assess the effect that missing data could have on our hypothesis. Firstly, a per protocol analysis was performed considering the participants who adhered to the research protocol. Subsequently, an intention to treat approach was used as main analysis to analyze participants according to the group to which they were originally assigned. If data were missing at 1^st ^and 2^nd ^follow-up (i.e. at 3 and 6 months), the way of dealing with missing data was substitution with the mean of the non-improved subjects in the GPR group and of the non-worsened subjects in the SE group. If a subject dropped out of the study after the 1^st ^follow-up, this value was carried forward and assumed to be the value of the 6-month follow-up.

The four outcome measures (RMDQ, ODI, FFT and VAS) were analyzed by a 2-way repeated-measures ANOVA with group (GPR and ST) and time (6-/3-month follow-up and baseline) as factors. An F-test for the interaction group × time was reported to test whether time affected the outcome differently in the two groups. One-way ANOVA was used to evaluate differences between groups at short- and mid-term follow-up for each outcome measure (differences were calculated as changes since baseline).

According to proposed cut-off values for minimal important change on frequently used measures of pain and functional status for LBP [38], a 30% change from baseline was considered a clinically meaningful improvement. The subjects were classified in three categories based on improvement on disability (as measured by RMDQ) and pain intensity (as measured by VAS): definitely improved (with a reduction of at least 30% on their RMDQ and VAS scores from baseline), possibly improved (with a reduction of at least 30% on their RMDQ score from baseline) and not improved. To evaluate the likelihood of improvement for the GPR group compared to the SE group, an ordered logistic regression model (adjusted for age, gender, BMI and white/blue-collar status) was performed using the classification into 3 ordered categories of improvement (from low to high) defined above as dependent variable. Odds ratio (OR) and 95% confidence interval (95% CI) were reported. Moreover, a multiple regression model (adjusted for age, gender, BMI and white/blue-collar status) of improvement (defined as the difference between the 2^nd ^follow-up and baseline) in the GPR group as compared to the SE group was performed for each outcome measure. The estimated β regression coefficients and 95% CI were calculated. To take into account the clustering of data, the robust cluster estimator of variance was used for all the regression models cited above.

Stata 9.0 SE software (Stata Corporation, Texas, TX) was used for all analyses, with significance set at *P *< 0.05.

## Results

From March 2008 to September 2009, 357 outpatients were evaluated by a clinician in the five centres included in this study: 187 in the four smaller centres and 170 in the largest one. In the four small centres, 137 patients were ineligible to enter the study on the basis of exclusion criteria. In particular, 33 were affected by acute or sub-acute LBP, 64 by specific causes of LBP (20 disc herniations, 20 lumbar stenoses, 4 spinal deformities, and 20 spondylolistheses), 2 by central or peripheral neurologic signs, and 2 by systemic illnesses. 36 patients who had undergone other physiotherapeutic interventions or surgical operations within 6 months prior to baseline assessment were also excluded. In the largest centre, 120 patients were excluded due to acute or sub-acute LBP in 10 cases, specific causes of LBP in 78 cases (15 disc herniations, 26 lumbar stenoses, 16 spinal deformities, and 21 spondylolistheses), central or peripheral neurologic signs in 5 cases, and systemic illnesses and psychiatric and mental deficits in 5 and 6 cases, respectively. 16 patients who had undergone other physiotherapeutic interventions or surgical operations within 6 months prior to baseline assessment were also excluded.

**Figure 4:**
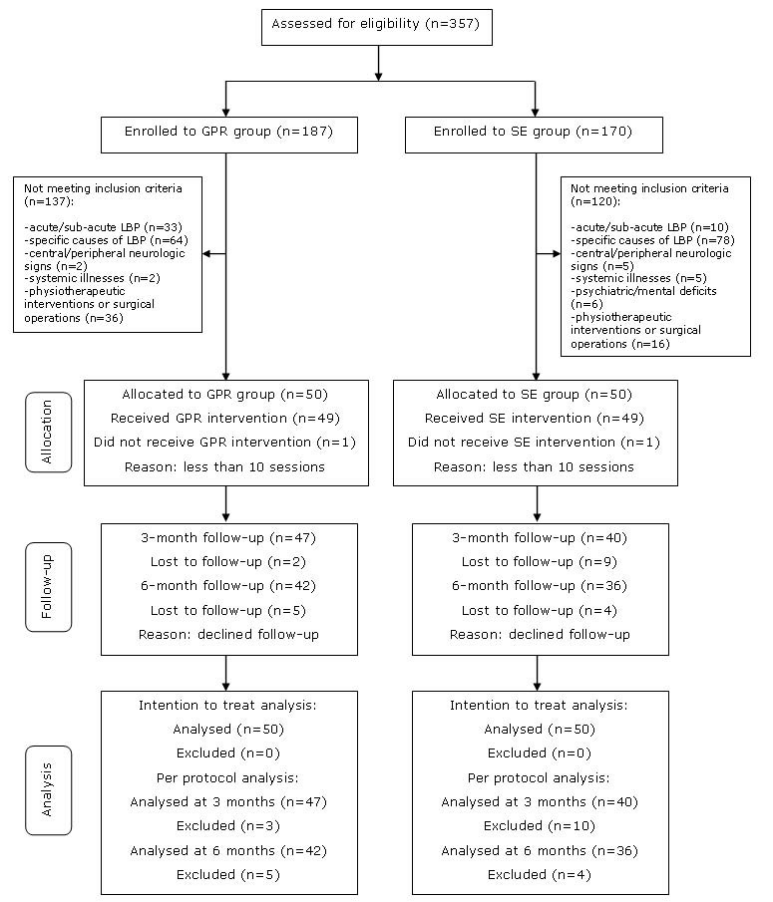
**Flow-chart of participants through the trial**. GPR Group: Global Postural Reeducation Group. SE Group: Stabilization Exercise Group.

The remaining 100 participants were enrolled: the 50 patients from the four smaller centres were allocated to the GPR group and the 50 patients from the largest centre were assigned to the SE group. 98 patients concluded the treatment session (one dropout for each group), then 87 participants completed the first follow-up, and 78 of them completed the study: 42 in the GPR group and 36 in the SE group (Figure 4). The baseline characteristics of participants did not differ significantly between the two groups, except for what concerned socioeconomic characteristics: blue-collar workers and subjects performing manual material handling were more frequent in the SE group (Table 1). Of note, no significant differences were detected between participants who were lost to follow-up and those who were followed-up, except for RMDQ in the GPR group and VAS in the SE group (Table 2).

**Table 1:** Baseline characteristics and outcome measures in the GPR and SE group.

	GPR Group (n = 50)	SE Group (n = 50)	*P*-value
Age (yr), mean (SD)	45.5 (12.2)	48.2 (13.2)	0.291^a^
BMI (kg/m^2^), mean (SD)	24.4 (3.2)	25.1 (3.3)	0.295^a^
Gender, n (%)			
Female	28 (56.0)	32 (64.0)	0.414^b^
Male	22 (44.0)	18 (36.0)	
Education level, n (%)			
Below high school diploma	3 (6.0)	10 (20.0)	0.073^b^
High school diploma	21 (42.0)	22 (44.0)	
University degree or higher	26 (52.0)	18 (36.0)	
Socio-occupational status, n (%)			
White-collar workers	43 (86.0)	31 (62.0)	0.006^b^
Blue-collar workers^c^	7 (14.0)	19 (38.0)	
Previous episodes of LBP in the last year, n (%)			
None	11 (22.0)	12 (24.0)	0.102^b^
1-2	28 (56.0)	18 (36.0)	
3-6	8 (16.0)	10 (20.0)	
>6	3 (6.0)	10 (20.0)	
Previous physical therapies, n (%)			
None	27 (54.0)	29 (58.0)	0.687^b^
At least one^d^	23 (46.0)	21 (42.0)	
Manual handling (load >10 Kg), n (%)			
No	43 (86.0)	34 (68.0)	0.032^b^
Yes	7 (14.0)	16 (32.0)	
Smoking status, n (%)			
Never	27 (54.0)	21 (42.0)	0.448^b^
Former	10 (20.0)	11 (22.0)	
Current	13 (26.0)	18 (36.0)	
Outcome measures			
RMDQ (0-24), mean (SD)	7.1 (5.3)	5.9 (4.6)	0.253^a^
ODI (0-100%), mean (SD)	22.5 (15.2)	22.2 (12.2)	0.908^a^
FFT (cm), mean (SD)	15.7 (12.5)	16.1 (12.9)	0.879^a^
VAS (0-100 mm), mean (SD)	60.9 (23.9)	57.7 (22.0)	0.485^a^

GPR Group: Global Postural Reeducation Group.

SE Group: Stabilization Exercises Group.

RMDQ: Roland and Morris Disability Questionnaire.

ODI: Oswestry Disability Index.

FFT: Fingertip-to-Floor Test.

VAS: Visual Analogue Scale as measure of low back pain (LBP).

^a^t-test.

^b^Χ^2 ^test.

^c^including housewives.

^d^between GPR method and standard physiotherapy.

**Table 2:** Baseline characteristics and outcome measures of completers and participants lost to 6-month follow-up in the GPR and SE group.

	GPR Group (n = 50)			SE Group (n = 50)		
	
	Completers (n = 42)	Lost to follow-up (n = 8)	*P*-value	Completers (n = 36)	Lost to follow-up (n = 14)	*P*-value
Age (yr), mean (SD)	44.7 (11.7)	49.6 (14.8)	0.301^a^	50.3 (12.5)	42.7 (13.7)	0.067^a^
BMI (kg/m^2^), mean (SD)	24.3 (3.5)	24.9 (1.4)	0.600^a^	25.1 (3.4)	25.1 (3.1)	0.999^a^
Gender, n (%)						
Female	25 (59.5)	3 (37.5)	0.277^b^	23 (63.9)	9 (64.3)	0.999^b^
Male	17 (40.5)	5 (62.5)		13 (36.1)	5 (35.7)	
Education level, n (%)						
Below high school diploma	2 (4.8)	1 (12.5)	0.137^b^	6 (16.7)	4 (28.6)	0.601^b^
High school diploma	20 (47.6)	1 (12.5)		17 (47.2)	5 (35.7)	
University degree or higher	20 (47.6)	6 (75.0)		13 (36.1)	5 (35.7)	
Socio-occupational status, n (%)						
White-collar workers	36 (85.7)	7 (87.5)	0.999^b^	23 (63.9)	8 (57.1)	0.659^c^
Blue-collar workers^d^	6 (14.3)	1 (12.5)		13 (36.1)	6 (42.9)	
Previous episodes of LBP in the last year, n (%)						
None	10 (23.8)	1 (12.5)	0.331^b^	8 (22.2)	4 (28.6)	0.739^b^
1-2	22 (52.4)	6 (75.0)		14 (38.9)	4 (28.6)	
3-6	8 (19.0)	0 (0.0)		8 (22.2)	2 (14.2)	
>6	2 (4.8)	1 (12.5)		6 (16.7)	4 (28.6)	
Previous physical therapies, n (%)						
None	21 (50.0)	6 (75.0)	0.261^b^	22 (61.1)	7 (50.0)	0.475^c^
At least one^e^	21 (50.0)	2 (25.0)		14 (38.9)	7 (50.0)	
Manual handling (load >10 Kg), n (%)						
No	37 (88.1)	6 (75.0)	0.310^b^	26 (72.2)	8 (57.1)	0.330^b^
Yes	5 (11.9)	2 (25.0)		10 (27.8)	6 (42.9)	
Smoking status, n (%)						
Never	21 (50.0)	6 (75.0)	0.587^b^	15 (41.7)	6 (42.9)	0.999^b^
Former	9 (21.4)	1 (12.5)		8 (22.2)	3 (21.4)	
Current	12 (28.6)	1 (12.5)		13 (36.1)	5 (35.7)	
Outcome measures						
RMDQ (0-24), mean (SD)	6.4 (5.0)	10.5 (5.9)	0.045^a^	5.3 (3.9)	7.6 (5.9)	0.103^a^
ODI (0-100%), mean (SD)	21.6 (13.9)	27.3 (21.6)	0.343	20.8 (11.6)	25.9 (13.3)	0.187^a^
FFT (cm), mean (SD)	15.4 (13.1)	17.6 (9.1)	0.643^a^	14.5 (12.3)	20.3 (14.0)	0.161^a^
VAS (0-100 mm), mean (SD)	60.3 (23.6)	64.4 (26.9)	0.660^a^	53.8 (22.3)	67.9 (18.1)	0.040^a^

GPR Group: Global Postural Reeducation Group.

SE Group: Stabilization Exercises Group.

RMDQ: Roland and Morris Disability Questionnaire.

ODI: Oswestry Disability Index.

FFT: Fingertip-to-Floor Test.

VAS: Visual Analogue Scale as measure of low back pain (LBP).

^a^t-test.

^b^Fisher's exact test.

^c^Χ^2 ^test.

^d^including housewives.

^e^between GPR method and standard physiotherapy.

The outcome measures at baseline and at short- and mid-term follow-up were reported in Table 3. Comparing the differences between groups at short- and mid-term follow-up, the GPR group revealed a significant reduction (from baseline) of all outcome measures with respect to the SE group, using the intention to treat approach as well as the per protocol analysis.

**Table 3:** Comparison between the GPR and SE group at short- and mid-term follow-up (ie 3 and 6 months).

	GPR Group (n = 50)		SE Group (n = 50)		
	
Intention to treat	mean (SD)	Change^a^ mean (SD)	mean (SD)	Change^a^ mean (SD)	*P*-value^b^
RMDQ (0-24)					
Baseline	7.1 (5.3)	--	5.9 (4.6)	--	
3 months	3.1 (3.9)	-4.0 (5.2)	5.6 (5.3)	-0.3 (4.1)	< 0.001
6 months	2.9 (3.5)	-4.2 (5.8)	5.4 (5.0)	-0.5 (2.9)	< 0.001
ODI (0-100%)					
Baseline	22.5 (15.2)	--	22.2 (12.2)	--	
3 months	12.2 (12.8)	-10.4 (15.0)	22.0 (15.6)	-0.2 (10.8)	< 0.001
6 months	12.3 (12.0)	-10.3 (17.9)	21.1 (15.4)	-1.1 (9.5)	0.002
FFT (cm)					
Baseline	15.7 (12.5)	--	16.1 (12.9)		
3 months	8.6 (9.4)	-7.2 (8.9)	15.4 (13.3)	-0.7 (11.8)	0.003
6 months	7.0 (9.1)	-8.8 (11.5)	13.5 (12.3)	-2.6 (12.2)	0.011
VAS (0-100 mm)					
Baseline	60.9 (23.9)	--	57.7 (22.0)	--	
3 months	23.9 (23.6)	-37.0 (29.4)	52.0 (25.0)	-5.7 (25.6)	< 0.001
6 months	31.5 (25.6)	-29.4 (31.8)	49.4 (24.8)	-8.3 (22.4)	< 0.001

Per protocol	mean (SD)	Change^a^ mean (SD)	mean (SD)	Change^a^ mean (SD)	*P*-value^b^

RMDQ (0-24)					
Baseline	7.1 (5.3)	--	5.9 (4.6)	--	
3 months	2.7 (3.5)^c^	-4.3 (5.2)^c^	6.0 (5.2)^d^	0.2 (4.4)^d^	< 0.001
6 months	2.6 (2.9)^e^	-3.8 (5.2)^e^	5.4 (4.8)^f^	0.1 (3.0)^f^	< 0.001
ODI (0-100%)					
Baseline	22.5 (15.2)	--	22.2 (12.2)	--	
3 months	11.0 (11.7)^c^	-11.4 (14.9)^c^	23.4 (16.1)^d^	1.3 (11.6)^d^	< 0.001
6 months	11.8 (11.0)^e^	-9.8 (16.4)^e^	20.9 (16.1)^f^	0.2 (10.8)^f^	0.003
FFT (cm)					
Baseline	15.7 (12.5)	--	16.1 (12.9)	--	
3 months	7.8 (9.0)^c^	-7.7 (9.0)^c^	15.8 (13.5)^d^	0.6 (12.9)^d^	< 0.001
6 months	6.1 (8.9)^e^	-9.3 (12.1)^e^	13.1 (12.2)^f^	-1.4 (13.6)^f^	0.008
VAS (0-100 mm)					
Baseline	60.9 (23.9)	--	57.7 (22.0)	--	
3 months	20.9 (20.8)^c^	-39.3 (28.7)^c^	51.1 (26.8)^d^	-3.0 (28.1)^d^	< 0.001
6 months	30.5 (24.0)^e^	-29.7 (30.7)^e^	48.2 (26.2)^f^	-5.6 (22.9)^f^	< 0.001

GPR Group: Global Postural Reeducation Group.

SE Group: Stabilization Exercises Group.

RMDQ: Roland and Morris Disability Questionnaire.

ODI: Oswestry Disability Index.

FFT: Fingertip-to-Floor Test.

VAS: Visual Analogue Scale as measure of low back pain (LBP).

^a^Change scores are changes since baseline, negative values indicate improvements.

^b^One-way ANOVA F-test.

^c^n = 47.

^d^n = 40.

^e^n = 42.

^f^n = 36.

Applying the intention to treat approach, a statistically significant group × time interaction was observed for RMDQ (F_2,196 _= 13.2, P < 0.001), ODI (F_2,196 _= 9.7, P < 0.001), FFT (F_2,196 _= 6.8, P = 0.001) and VAS (F_2,196 _= 19.7, P < 0.001), suggesting that the two groups were changing over time, but in different ways. Statistically significant group × time interactions were also observed for each outcome analysed with the per protocol approach (data not shown).

**Figure 5:**
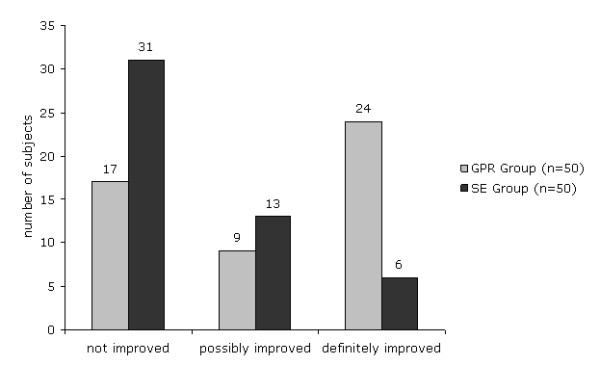
**Distribution of definitely improved, possibly improved and not improved subjects in the GPR and SE group**. Definitely improved: reduction of at least 30% on RMDQ and VAS scores from baseline. Possibly improved: reduction of at least 30% on RMDQ score from baseline.

Figure 5 reports the distribution of subjects according to three categories based on improvement on disability (as measured by RMDQ) and pain intensity (as measured by VAS). Of note, 48% of the subjects in the GPR group definitely improved compared to 12% in the SE group, whereas 34% of the subjects in the GPR group did not improve compared to 62% in the SE group. Furthermore, the ordered logistic regression model showed an increased likelihood of definitive improvement (at least 30% reduction on RMDQ and VAS scores from baseline) for the GPR group compared to the SE group (OR 3.9, 95% CI 2.7 to 5.7).

The multiple regression models showed significant differences in improvement (defined as the difference between the 2^nd ^follow-up and baseline) in the GPR group compared to the SE group for each outcome measure evaluated, apart from FFT. The four models reported the following results: RMDQ (β = -3.6, 95% CI -4.8 to -2.4), ODI (β = -8.8, 95% CI -11.4 to -6.3), FFT (β = -5.2, 95% CI -11.8 to 1.3) and VAS (β = -22.2, 95% CI -28.4 to -16.1).

## Discussion

To our knowledge, this is the first controlled study comparing a GPR program to a physical therapy SE program in patients with persistent LBP. Our findings support the hypothesis that the GPR intervention (specifically the posture adopted) is effective in treating persistent LBP with low disability levels, when compared to SE. Patients allocated to the GPR group showed significant improvement in functional disability and pain intensity as compared to the SE group. Of note, the improvement obtained at short-term follow-up was maintained at mid-term follow-up for each outcome.

Our results are similar to those obtained in two RCTs by Fernandez-de-las-Peñas et al. [19,20], who showed better results of the GPR as compared to a program of analytical exercises in patients with ankylosing spondylitis, and to those obtained in other studies on LBP cited in the review by Vanti et al. [18]. Therefore, it seems that global reeducation is more effective in reducing pain and disability in subjects with LBP than segmental techniques. However, these results are different from those by Cunha et al. [25], who did not find different outcomes comparing conventional static stretching and muscle chain stretching in chronic neck pain. The reasons for these differences may be related both to the areas affected by spinal pain, and to the fact that GPR might be more effective when compared to analytical stabilization or mobilization techniques, although not superior to other stretching techniques.

When considering the clinical impact of our research [38], we can state that the GPR program produced a clinically meaningful improvement. In fact, 48% of subjects in the GPR group obtained a reduction of at least 30% in their RMDQ and VAS scores, compared to the 12% in the SE group. It should be noted that our results seem to demonstrate the effectiveness of the GPR program at relatively low disability levels.

In our study, the SE group obtained a slight improvement in functional disability, pain intensity and mobility of the whole spine and pelvis at the 3- and 6-month follow-up. The mean effects of the SE program were less relevant than those reported in some previous trials [39,40], but are in line with those obtained in some other recent studies [12,41]. An important aspect of the lack of agreement among these studies is the absence of subjects' subgrouping. According to Hayden et al., clinical trials should investigate the effectiveness of specific exercises in well-defined LBP subgroups [42]. However, the identification of subgroups is a difficult process, since it cannot yet be guided by a coherent theory of causation of back pain [13]. Moreover, the disability levels of the subjects included in our study are relatively low compared to previously published disability scores in 'chronic LBP' populations.

The main strength of our study was that patients undergoing a specific program were unaware of the presence of another training, because patients recruited by the same centre underwent the same treatment approach. Moreover, both treatments consisted of a one-to-one supervised exercise program actively involving the patient: according to the literature, these kinds of management are effective to reduce disability and improve function in chronic LBP [43-45].

Apart from the robust clustering, analysis could be adjusted only for patient factors (e.g. because of the differences in socioeconomic factors between patient arms we adjusted for socio-occupational status). On the other hand, there are other factors that might possibly explain, at least in part, the differences in outcome measures. Considering the differences in the physical therapists' experience, it has to be underlined that the more experienced physical therapists were those involved in the SE program. It is generally assumed that greater experience is associated with better clinical outcomes [46-48]. However, some recent studies evaluating the relationship between physiotherapists' years of experience and patient outcomes reported that years of experience were not associated with improved patient outcomes in outpatient rehabilitation [49,50]. Moreover, in our opinion the size of the centers does not affect care quality, since this is strictly related to the close relation between patient and physical therapist.

The main limitation of this study was the absence of randomization. This practical choice could have led to some potential sources of bias: the two groups were similar at baseline, but slightly differed for socio-economical characteristics; this issue was considered in the multivariate analysis, taking also into account the possible different aggregation of patients within the different centres. The absence of randomization could have also influenced the patient's allocation to a specific physical therapist. Therefore, the possible influence of each physical therapists' experience and the psychosocial aspects of the patient-physical therapist relationship must be considered, as some physical therapists could have had the capabilities to establish a better relationship with their patients, thus positively influencing the effects produced by the treatment and the motivation for self-management.

Another important limitation was the elevated number of dropouts in the SE group (28%). Furthermore, dropped-out patients differed on critical baseline characteristics (RMDQ and VAS) from those who completed the study; on the other hand, they were equally represented in the two groups (SE and GPR). The high number of dropouts was mainly due to the determination of patients to give up treatment in the absence of expected results. This aspect was managed with the intention to treat approach, which confirmed the improvement obtained in the GPR group with respect to the SE group at the per protocol analysis.

Moreover, patients' adherence to the home exercise program could not be monitored. Indeed, adherence to the therapeutic program represents a crucial aspect of chronic LBP treatment [51,52] and seems to be related to professional behaviors and explanations and to the total number of exercises prescribed [53,54], as well as to individual and psychological characteristics [8]. Finally, since the possible influence of physical and sport activities or cognitive-behavioral aspects was not considered, the presence of a complicated condition as a result of physical and psychosocial factors cannot be excluded (yellow flags) [45].

As a consequence, although we cannot definitely state that GPR alone is effective for patients with chronic LBP, GPR can be considered an important approach in the management of patients with persistent LBP and low disability levels. Because dropouts from both groups had higher levels of pain and disability than the subjects who completed the trial, we cannot apply our results to subjects with chronic, more disabling LBP.

Several physical therapy methods appeared effective in LBP; most of them have been studied by rigorous clinical trials. The recent trend of research on this topic allows to identify clinical prediction rules that can be applied to LBP subgroups. Some of the proposed classifications for subgrouping patients with LBP are related to techniques as manipulation, stabilization, specific exercises, or traction. Global postural treatment is not considered within that subgrouping. This is mainly due to a general lack of knowledge and to the little evidence of effectiveness of this method. Our study may represent the first step in this direction, but it should be followed by further, higher level studies. Accordingly, some suggestions for future research are the measurement of the clinical effects of the GPR on specific LBP subgroups, with respect to the age of patients, the phase of disorder (e.g. acute, subacute or chronic pain), and the clinical characteristics. Moreover, the effectiveness of GPR should be compared with other techniques, as manipulative therapy, cognitive behavioral therapy, etc.

## Conclusions

In conclusion, this study showed a significant improvement on disability and intensity of pain employing a GPR program, using these three postures in particular, as compared to a conventional physical therapy regimen, in patients with persistent LBP. These results appeared significant both at short- and at mid-term follow-up. This study was performed in outpatient physical therapy centres, and its results could be generalized to groups of patients with similar characteristics (i.e., patients with chronic LBP and low disability levels, seeking care for their LBP problems). However, these results must be taken with caution, considering the potential confounding role of some factors, in particular the patient drop out rate and the absence of randomization. Future research may identify which groups of patients could better respond to GPR treatment, similarly to what has already been done for other therapeutic procedures. Therefore, these encouraging results must be confirmed by further studies with higher methodological standards, including randomization, larger sample size, longer follow-up and subgrouping of LBP subjects.

## Competing interests

The authors declare that they have no competing interests.

## Authors' contributions

FB and PP designed the study. FB was responsible for data collection. SC and SM were responsible for data analysis, together with FB and PP. FB, SC, SM and PP contributed to interpretation of data, together with RM, CV and FSV. FB, SC, SM and PP drafted the manuscript, together with RM and CV. All authors critically revised the manuscript. All authors read and approved the final manuscript.

## Pre-publication history

The pre-publication history for this paper can be accessed here:

http://www.biomedcentral.com/1471-2474/11/285/prepub
